# Predicting intradialytic hypotension using heart rate variability

**DOI:** 10.1038/s41598-019-39295-y

**Published:** 2019-02-22

**Authors:** Samel Park, Wook-Joon Kim, Nam-Jun Cho, Chi-Young Choi, Nam Hun Heo, Hyo-Wook Gil, Eun Young Lee

**Affiliations:** 1 Department of Internal Medicine, Soonchunhyang University Cheonan Hospital, Cheonan, Korea; 2 Department of Biostatistics, Soonchunhyang University Cheonan Hospital, Cheonan, Korea; 3 Institute of Tissue Regeneration, College of Medicine, Soonchunhyang University, Cheonan, Korea

## Abstract

This study aimed to identify whether a new method using heart rate variability (HRV) could predict intradialytic hypotension (IDH) for one month in advance for patients undergoing prevalent hemodialysis. A total 71 patients were enrolled, and baseline clinical characteristics and laboratory results were collected when HRV was measured, then, the frequency of IDH was collected during the observation period. HRV parameters included heart rate, R-R interval, the standard deviation of N-N interval, the square root of the mean squared differences of successive NN intervals, very low frequency, low frequency, high frequency, total power, and low frequency/high frequency ratio. During the one-month observation period, 28 patients experienced 85 cases of IDH (10.0% of a total 852 dialysis sessions). Among the clinical and laboratory parameters, ultrafiltration rate, prior history of diabetes, coronary artery disease, or congestive heart failure, age, intact parathyroid hormone level, and history of antihypertensive drug use were integrated into the multivariate model, referred to as a basic model, which showed significant ability to predict IDH (the area-under-curve [AUC], 0.726; *p* = 0.002). In HRV parameters, changes between the early and middle phases of hemodialysis (referred to Δ) were identified as significant independent variables. New models were built from the combination of Δ values with the basic model. Among them, a model with the highest AUC value (AUC, 804; *p* < 0.001) was compared to the basic model and demonstrated improved performance when HRV parameters were used (*p* = 0.049). Based on our results, it is possible that future IDH might be predicted more accurately using HRV.

## Introduction

Intradialytic hypotension (IDH), a very challenging complication in patients on hemodialysis, is associated with increased mortality^1,2^, cerebral ischemia^3^, vascular access thrombosis^4^, cardiovascular events^5^, and hospitalizations^6^. The prevalence of IDH has been reported to differ widely, because previous studies were based on varying definitions of IDH^7^. Recently, Flythe *et al*. showed that the prevalence of IDH defined by K/DOQI classification was 9.6%^8^. Numerous risk factors of IDH have been reported, including older age, female sex, Hispanic ethnicity, longer dialysis vintage, diabetes mellitus, coronary artery disease, left ventricular hypertrophy, ingestion of a meal before hemodialysis, increased body mass index, lower albumin levels, and higher interdialytic weight gain^9^.

The mechanism of IDH is intricate; however, intravascular hypovolemia developed during hemodialysis is suspected to be the main etiologic cause. Several compensatory mechanisms, including cardiac responses to maintain cardiac output and venous return, arteriolar vasoconstriction to increase total peripheral resistance, and plasma refilling from the interstitial and intracellular compartments, are activated if intravascular hypovolemia develops^10^. The dysfunction of the autonomic nervous systems is also pivotal in developing IDH. In a previous study, Kersh *et al*. showed that IDH developed because of autonomic nervous insufficiency. In patients with normal autonomic function, a rise in systemic resistance and in heart rate was observed, but in those with autonomic dysfunction, total systemic resistance fell during IDH although heart rate was fixed^11^. Converse *et al*. reported that hemodialysis-induced hypotension is caused by autonomic dysfunction, leading to an inadequate sympathetic response to hypovolemia developed during hemodialysis^12^.

Heart rate variability (HRV) provides a non-invasive and reliable way to measure autonomic dysfunction^13^. In normal individuals, heart rate fluctuates with respiration because of a higher degree of beat-to-beat variability. An individual with higher HRV implies a functionally adequate autonomic function^14^. A study to evaluate HRV during hemodialysis showed that the sympathetic response is activated and increases during hemodialysis; however, in patients prone to IDH, such activation is impaired in the late phase of dialysis, contributing to development of IDH^15^. Another study that measured HRV during hemodialysis showed that sympathetic nervous activity increases; however, the increased tone of the sympathetic nervous system suddenly falls when symptomatic hypotension develops (“Bezold-Jarisch reflex”)^16^. Recently, it was also reported that HRV is a useful indicator for IDH^17^. Given the previous studies, we hypothesized that HRV is a reliable way to predict the development of future IDH.

## Materials and Methods

This study was conducted in accordance with the Declaration of Helsinki, and the protocol was approved by the Institutional Review Board of Soonchunhyang University Cheonan Hospital (IRB No. 2017-02-008-007). All patients provided their written informed consent.

### Patient selection and collection of clinical and laboratory data

The patients on prevalent hemodialysis in Soonchunhyang University Cheonan Hospital Artificial Kidney Center (Cheonan, South Korea) were recruited, and 71 patients participated in this prospective observational study. All patients were older than 18 years, had undergone thrice-weekly hemodialysis for at least three months, and had not been hospitalized for acute illness during the preceding month. Patients with atrial fibrillation, allergy to stitch agent, or short life expectancy because of chronic illness, such as terminal cancer, were excluded.

The patient’s age, sex, prior history of diabetes, coronary artery disease, and congestive heart failure, leading cause of end-stage renal disease (ESRD), dialysate temperate, vintage of dialysis, ultrafiltration rate, complete blood counts, blood chemistry, electrolyte, uric acid, intact parathyroid hormone, Kt/V, urea reduction rate, and normalized protein catabolic rate were collected when HRV was measured. The history of antihypertensive agents (angiotensin II receptor blocker or angiotensin-converting-enzyme inhibitor, calcium channel blocker, and β-blocker) was also investigated.

### Monitoring of intradialytic hypotension

We defined IDH following K/DOQI guidelines as: a decrease in systolic blood pressure of 20 mm Hg or more or a decrease in mean arterial pressure of 10 mm Hg or more; the presence of symptoms of end-organ ischemia; and a need for intervention carried out by the dialysis staff^18^. The total number of IDH events was collected for 1 month during the 12 sessions for each patient. The interventions done to restore blood pressure were collected when IDH developed. Vital signs were measured hourly during hemodialysis if the patients did not complain about any symptoms. When the patients did complain, the nursing staff immediately checked the vital signs and judged whether the symptoms were those of IDH.

### Measurement of heart rate variability

HRV data were measured using a T-REX (Taewoong Medical Co., Ltd, Seoul, South Korea), which is a portable ECG monitoring device dedicated to HRV analysis. The device is extremely small and lightweight (only 10 grams), so the patients had little discomfort wearing it during hemodialysis. It is more robust against motion artifacts than is a conventional Holter monitor^19^. The T-REX was attached to the patient’s anterior chest. After attachment, the patients rested for ten minutes to minimize other effects, such as heart rate changes during the walk into dialysis centers and from position changes. For an accurate recording of HRV data, all patients were requested to stay as supine as possible during hemodialysis. HRV measurements were started with the initiation of hemodialysis.

After hemodialysis, the recorded ECG signal was processed. R peaks were automatically detected by software and visually inspected by experienced researchers. After R-R interval (RRI) was calculated, RRIs more than 20% different from previous ones were removed as ectopic beats^20^. HRV parameters were generated following the recommendations of the Task Force of the European Society of Cardiology and the North American Society of Pacing and Electrophysiology^13^. The mean heart rate (HR), the standard deviation of N-N interval (SDNN), and the square root of the mean squared differences of successive NN intervals (RMSSD) were measured for the time-domain analysis. The frequency-domain analysis was performed using a Welch periodogram with 4 Hz resampling with linear interpolation, 64-second windows and 75% overlap^21^. The adopted parameters and frequency bands for each were very low frequency (0.003 to 0.04 Hz, VLF), low frequency (0.04 to 0.15 Hz, LF), high frequency (0.15 to 0.40 Hz, HF), total power (TP), and LF/HF ratio.

All HRV data collected for 240 minutes were divided into five-minute intervals, giving a total of 48 segments. For statistical analysis, we selected three periods to cover the early, middle, and late phases. Because of the high variability of HRV, each phase consisted of two consecutive segments (a total of 10 minutes per phase) and the average value of the two segments was used in the final variables. In the pattern we ended up with, the early phase used segments 1 and 2, the middle phase used segments 24 and 25, and the late phase used segments 47 and 48, out of the original 48 segments.

### Statistical method

Continuous data were expressed as mean ± standard deviation or median (25^th^–75^th^ percentile) as appropriate. Categorical data were expressed by frequency (proportions, %). A chi-squared test or Fisher’s exact test was used for categorical data as appropriate. Continuous variables between the two groups were compared by Student’s *t*-test or Mann-Whitney U test as appropriate. We looked for statistically significant differences in continuous data between pre- and post-hemodialysis by using a paired t-test or Wilcoxon signed-rank test as appropriate. Repeated-measures analysis of variance or a Friedman test was used to explore whether there were significant changes in HRV parameters between phases.

Univariate and multivariate analysis using a negative binomial model was used to explore independent risk factors for IDH. Negative binomial analysis was used because the study had only a few patients and faced over-dispersion. The variables with a *p* value < 0.10 in the univariate model were integrated into the multivariate model, in which the frequency of IDH was used as an independent variable, and the primary outcome was the occurrence of IDH. When creating a multivariable model, concerns about multi-collinearity arose. To handle these concerns and select the most valuable variables to be used in the model, we built various models and calculated the area-under-curve (AUC) values of the models and the variance inflation factor (VIF) values of parameters which were included in each separate model. Models with variables having VIF values greater than 10, and those with variables found to have mutually strong correlations were considered to have multi-collinearity and were excluded.

To estimate the goodness of fit, the Akaike Information Criterion and the Bayesian Information Criterion were used. The predicted value of the linear predictor of the model was used as a variable for the area-under-curve (AUC) calculation. The receiver operating characteristic (ROC) curves were drawn and compared between two models using DeLong’s method. Statistical analyses were performed using SPSS 22.0 for Windows (SPSS, Inc., Chicago, IL, USA) and R software (Vienna, Austria, version 3.4.3).

## Results

During the one-month observation period, among the total 71 patients, 28 experienced at least one event of IDH (IDH group), but the others (non-IDH group) had not. The patients in the IDH group had 2.0 (1.0–3.8) IDH experiences during the month. Table 1 shows the clinical and laboratory characteristics of the two groups. The post-dialysis systolic and diastolic blood pressure in patients in the non-IDH group was higher than for those in the IDH group (Table 1). There was no other statistically significant difference between the two groups. For the patients in the IDH group, the post-dialysis systolic blood pressure was significantly lower than the pre-dialysis systolic blood pressure (124 ± 23 *vs*. 141 ± 23, *p* < 0.001), but this result was not observed for the non-IDH group (141 ± 21 *vs*. 136 ± 17, *p* = 0.323).

**Table 1:** Baseline characteristics of the patients in IDH and non-IDH groups.

		non-IDH (*n* = 43)	IDH (*n* = 28)	*p* value
Age, year		53.2 ± 12.7	57.3 ± 13.3	0.200
Prevalence of IDH, /month		0	2.0 (1.0–3.8)	
Male, n (%)		22 (51.2)	18 (64.3)	0.276
DM, n (%)		28 (65.1)	16 (57.1)	0.499
CAD, n (%)		3 (7.0)	6 (21.4)	0.141
CHF, n (%)		3 (7.0)	6 (21.4)	0.141
Low dialysate temp., n (%)		4 (9.3)	5 (17.9)	0.304
Cause of ESRD, n (%)	HTN	11 (25.6)	12 (42.9)	0.481
	DM	11 (25.6)	6 (21.4)	
	CGN	18 (41.9)	8 (28.6)	
	PCKD	3 (7.0)	2 (7.1)	
Vintage of dialysis, months		57.0 (33.0–125.0)	82.5 (32.3–158.0)	0.434
Ultrafiltration rate, kg		2.66 ± 1.19	3.09 ± 0.95	0.113
pre-dialysis	SBP^†^, mmHg	136 ± 17	141 ± 23^*^	0.323
	DBP^†^, mmHg	77 ± 12	77 ± 13	0.906
	HR^†^, /min	73 ± 14	76 ± 8	0.251
post-dialysis	SBP^†^, mmHg	141 ± 21	124 ± 23^*^	0.001
	DBP^†^, mmHg	80 ± 13	74 ± 11	0.040
	HR^†^, /min	75 ± 13	79 ± 12	0.246
ARB or ACEI, n (%)		34 (79.1)	15 (53.6)	0.023
CCB, n (%)		29 (67.4)	12 (42.9)	0.040
β-blocker, n (%)		21 (48.8)	14 (50.0)	0.924
White blood cells, count/μL		5600 (4430–7090)	5725 (4458–6678)	0.977
Hemoglobin, g/dL		10.8 ± 1.2	10.4 ± 1.3	0.285
Hematocrit, %		31.6 ± 3.9	30.4 ± 4.0	0.223
Platelet, x1000/μL		171 ± 36	184 ± 60	0.316
Protein, g/dL		6.7 ± 0.4	6.8 ± 0.5	0.568
Albumin, g/dL		3.8 (3.7–4.1)	3.8 (3.6–4.0)	0.300
Glucose, mg/dL		94 (79–135)	122 (77–166)	0.356
Blood urea nitrogen, mg/dL		60.6 (52.7–70.3)	63.0 (58.0–73.2)	0.188
Creatinine, mg/dL		9.7 ± 3.1	10.6 ± 2.6	0.234
Alkaline phosphatase, IU/L		63 (48–73)	63 (55–82)	0.724
Sodium, mmol/L		139 ± 2	139 ± 3	0.246
Potassium, mmol/L		5.1 ± 0.7	4.9 ± 0.5	0.207
Chloride, mmol/L		99 ± 4	98 ± 4	0.075
tCO2, mmol/L		22.1 ± 2.6	22.2 ± 3.7	0.903
Uric acid, mg/dL		6.8 (6.0–8.0)	7.7 (6.0–8.6)	0.141
Calcium, mg/dL		8.8 (8.3–9.2)	8.9 (8.4–9.3)	0.689
Phosphorus, mg/dL		4.2 (3.4–5.2)	4.5 (3.5–5.2)	0.728
Ca x P		37.7 ± 15.7	38.9 ± 11.0	0.723
intact PTH, pg/mL		254.4 (124.3–390.9)	202.2 (121.4–280.1)	0.188
Dialysis adequacy (Kt/V)		1.856 ± 0.369	1.769 ± 0.287	0.293
Urea reduction rate, %		79.4 (70.6 ± 82.0)	76.0 (71.0–78.9)	0.228
nPCR		0.880 (0.799–1.068)	0.918 (0.824–1.026)	0.638

^*^Significant difference between pre-dialysis value and post-dialysis value.

^†^Data were collected before initiation and after finish hemodialysis.

Abbreviation: IDH, intradialytic hypotension; DM, diabetes mellitus; CAD, coronary artery disease; CHF, congestive heart failure; temp., temperature; ESRD, end-stage renal disease; HTN, hypertension; CGN, chronic glomerulonephritis; PCKD, polycystic kidney disease; SBP, systolic blood pressure; DBP, diastolic blood pressure; HR, heart rate; ARB, angiotensin II receptor blocker; ACEI, angiotensin-converting-enzyme inhibitor; CCB, calcium channel blocker; tCO2, total carbon dioxide; Ca x P, calcium phosphorus product; PTH, parathyroid hormone; nPCR, normalized protein catabolic rate.

Altogether, patients received a total of 852 dialysis sessions, and 85 cases (10.0%) of IDH had occurred (Table 2). The initial therapy to restore blood pressure was to change the patients to the Trendelenburg position, which was applied to all cases of IDH. Among these, 24 cases of IDH were recovered by the Trendelenburg position only. However, in the others, additional procedures were needed for recovery. After the Trendelenburg position, reduction of blood flow rate was the next most-frequent approach to achieve recovery (Table 2).

**Table 2:** The number of intradialytic hypotension and interventions to restore blood pressure.

No. of events	
Patients with IDH	28
A total of hemodialysis	852
Events of IDH	85
**Intervention for IDH**	
Leg elevation only	24
with additional treatment	61
Saline infusion	10
Reduction of BFR	56
Reduction of UFR	14
Early termination	7

Abbreviation: No., number; IDH, intradialytic hypotension; BFR, blood filtration rate; UFR, ultrafiltration rate.

**Figure 1:**
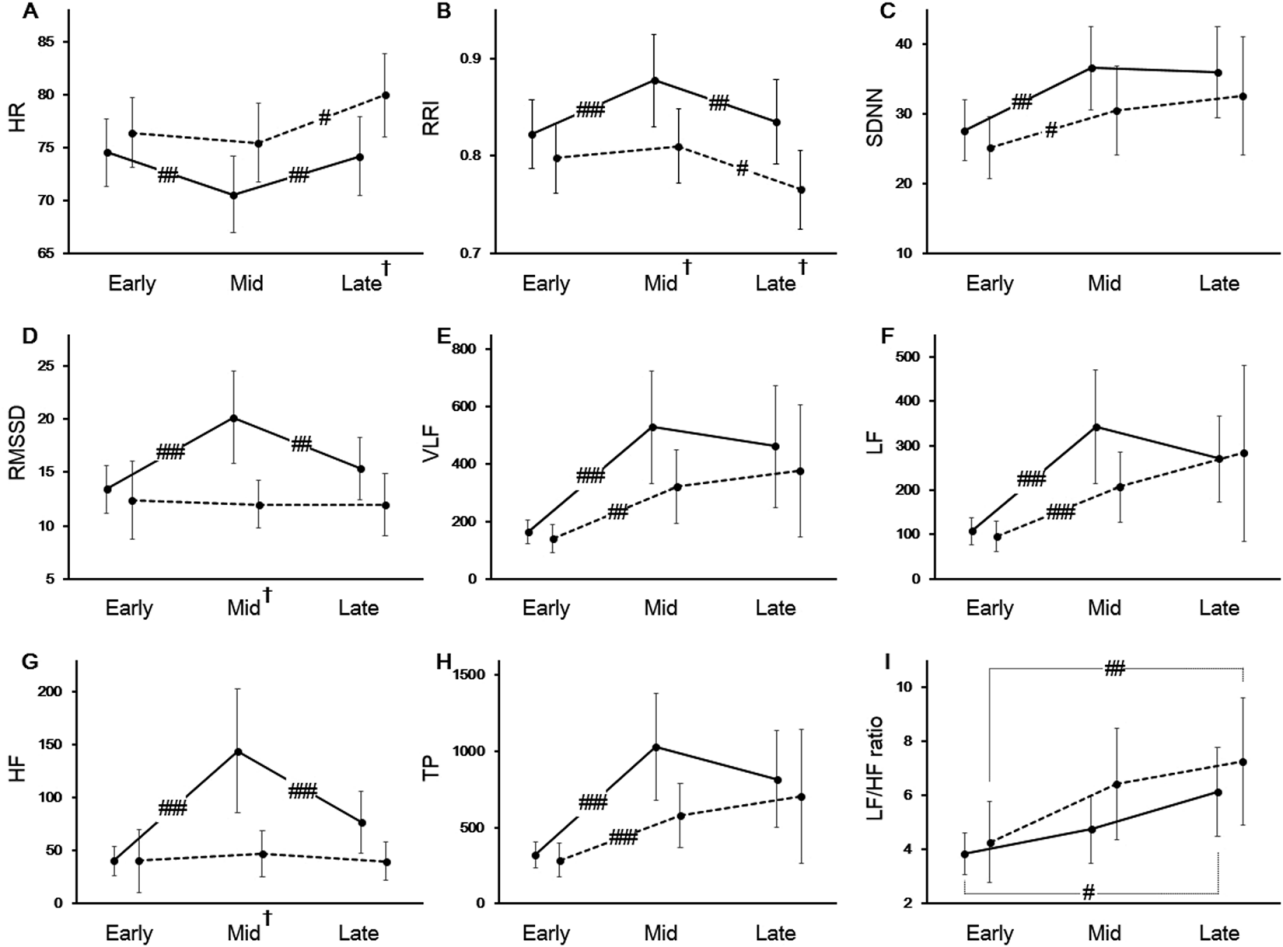
Changes of heart rate variability during hemodialysis. (**A**) Heart rate (HR), (**B**) R-R interval (RRI), (**C**) the standard deviation of N-N interval (SDNN), (**D**) the square root of the mean squared differences of successive NN intervals (RMSSD), (**E**) very low frequency (VLF), (**F**) low frequency (LF), (**G**) high frequency (HF), (**H**) total power (TP), and (**I**) LF/HF ratio. Solid line, non-IDH group; dashed line, IDH group; ^#^*p* < 0.05; ^##^*p* < 0.01; ^###^*p* < 0.001, between the two phases. ^†^*p* < 0.05, between the two groups.

Figure 1 shows the changes of HRV parameters between the phases, that is, between the early and middle phases, and between the middle and late phases. In the non-IDH group, HR significantly reduced and then increased. In the case of RRI, a reversed pattern was observed. In the IDH group, the changes of HR and RRI were observed only between the middle phase and late phase. (Fig. 1A,B). At the late phase, HR in patients in the IDH group was higher than in those in the non-IDH group (Fig. 1A); however, RRI pattern was *vice versa* (Fig. 1B). A similar change pattern for SDNN, VLF, LF, and TP was observed. They increased significantly from the early to the middle phase in both groups (Fig. 1C,E,F,H). RMSSD and HF in patients in the non-IDH group significantly increased, then decreased; however, these changes were not observed in those in the IDH group (Fig. 1D,G). LF/HF ratio during hemodialysis gradually increased in both groups from the early phase to the late one: from 2.9 (1.9–5.1) to 4.0 (2.6–7.5) in the non-IDH group (*p* = 0.023) and from 3.2 (1.6–5.2) to 5.7 (2.2–9.4) in the IDH group (*p* = 0.002) (Fig. 1I).

Significant changes in the HRV parameters during hemodialysis were mainly observed between the early and middle phases; hence, these changes (i.e., the delta values) were used as new variables to make models. The results of the univariate model using these new variables are reported in Table 3. Among the clinical and laboratory data, variables with *p* < 0.10 in the univariate negative binomial models (the prior history of diabetes, older age, ultrafiltration rate, and intact parathyroid hormone), and the prior history of coronary artery disease, and congestive heart failure (which were considered to be important risk factors for IDH, even though their *p*-values was 0.225 and 0.846, respectively) are also reported in Table 3. Results from the history of antihypertensive agents are also displayed.

**Table 3:** Univariate negative binomial models for predicting intradialytic hypotension.

Variables	p value	Exp(B)	
with DM	0.015	2.22	(1.16–4.22)
with CAD	0.225	1.72	(0.72–4.14)
with CHF	0.846	1.13	(0.33–3.88)
Age, /10 years	0.052	1.30	(1.00–1.70)
UFR, /kg	0.008	1.57	(1.12–2.19)
iPTH, /100 pg/mL	0.026	0.78	(0.62–0.97)
ARB or ACEI	0.145	0.61	(0.32–1.18)
CCB	0.040	0.51	(0.27–0.97)
β-blocker	0.988	1.00	(0.53–1.89)
ΔHR	0.003	1.08	(1.03–1.14)
ΔRRI, /0.1 msec	<0.001	0.44	(0.27–0.72)
ΔSDNN	0.011	0.97	(0.94–0.99)
ΔRMSSD	0.002	0.91	(0.85–0.96)
ΔVLF, /100 msec^2^	0.004	0.84	(0.75–0.95)
ΔLF, /100 msec^2^	0.002	0.68	(0.53–0.87)
ΔHF, /100 msec^2^	0.003	0.40	(0.22–0.73)
ΔTP, /100 msec^2^	<0.001	0.86	(0.79–0.94)
ΔLF/HF ratio	0.511	1.03	(0.95–1.11)

Abbreviation: DM, diabetes mellitus; CAD, coronary artery disease; CHF, congestive heart failure; UFR, ultrafiltration rate; iPTH, intact parathyroid hormone; ARB, angiotensin II receptor blocker; ACEI, angiotensin-converting-enzyme inhibitor; CCB, calcium channel blocker; HR, heart rate; RRI, R-R interval; SDNN, the standard deviation of N-N interval; RMSSD, the square root of the mean squared differences of successive NN intervals; VLF, very low frequency; LF, low frequency; HF, high frequency; TP, total power.

Δ represents changes between the early and the middle phase.

**Figure 2:**
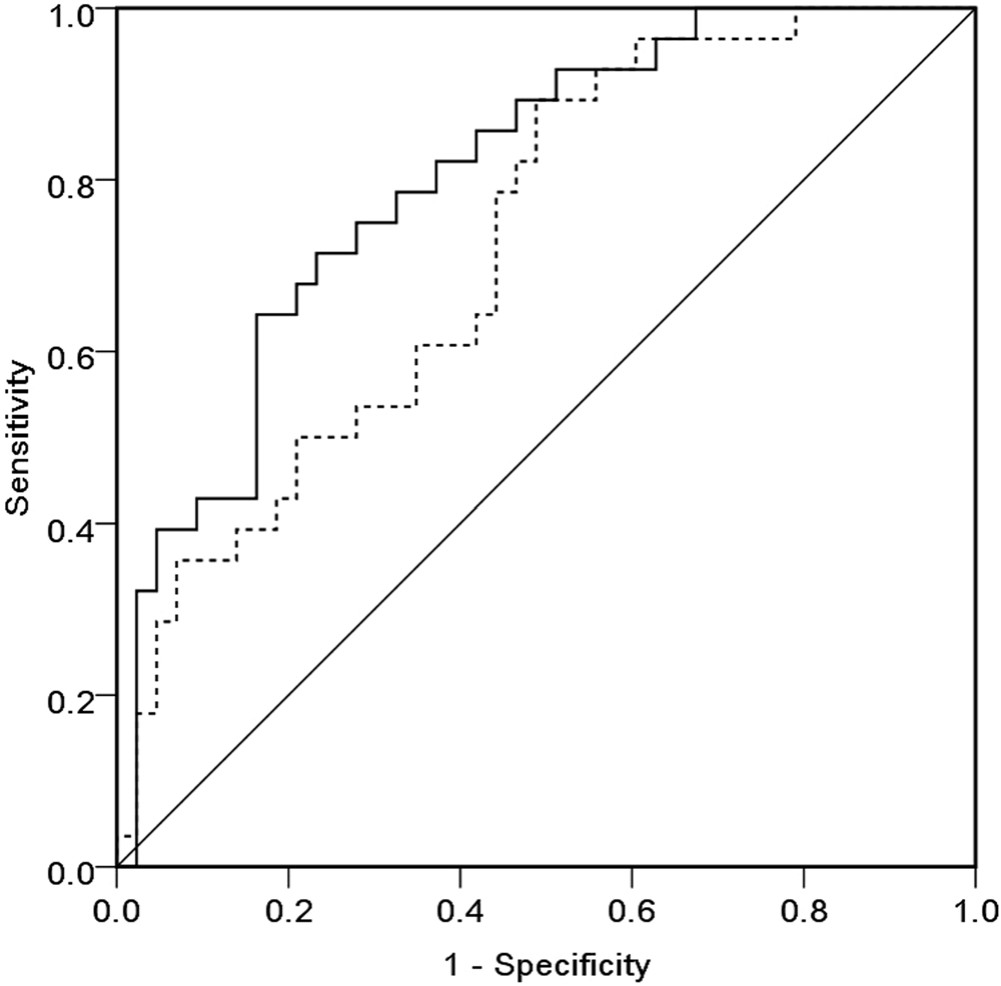
Receiver operating characteristic curves of Model 1 and Model 2 for predicting intradialytic hypotension. Dashed line, Model 1; Solid line, Model 2.

Then, multivariate negative binomial models were made using only the variables from clinical and laboratory data without any HRV results, leading to the basic model, in which the prior history of diabetes, coronary artery disease, or congestive heart failure, age, ultrafiltration rate, intact parathyroid hormone, and the history of antihypertensive agents were included (Model 1 in Table 4). After that, several combinations of models were created by integration of HRV parameters into the basic model. Among these models, five models with the highest AUC value are reported in order in Table 4. Model 2 had the highest AUC value (0.804), and the ROC curves of the basic model (Model 1 in Table 4) and Model 2 were drawn (Fig. 2). There was a significant difference between the two models (*p* = 0.049), which showed that adding the HRV parameters to the basic model significantly increased its power to predict IDH.

**Table 4:** Hazard ratios and goodness of fit of multivariate negative binomial models for predicting intradialytic hypotension.

	Model 1	Model 2	Model 3	Model 4	Model 5	Model 6
with DM	1.90	1.14	1.17	1.15	1.49	1.16
with CAD	0.75	1.01	0.98	0.98	1.09	1.00
with CHF	1.79	2.07	2.16	2.09	1.71	2.16
Age, /10 year	1.34	1.19	1.19	1.19	1.19	1.18
UFR, /kg	1.67^c^	1.66^c^	1.68^c^	1.68^c^	1.65^c^	1.66^c^
iPTH, /100 pg/mL	0.81	0.85	0.85	0.85	0.85	0.85
ARB or ACEI	0.52	0.40	0.39	0.39	0.46	0.40
CCB	0.55	0.71	0.73	0.73	0.66	0.70
β-blocker	1.27	1.10	1.07	1.08	1.04	1.09
ΔHR			1.04	1.04		
ΔRRI, /0.1 msec		0.65			0.57	0.65
ΔSDNN						
ΔRMSSD						
ΔVLF, /100 msec^2^						
ΔLF, /100 msec^2^			0.96			0.96
ΔHF, /100 msec^2^		0.87	0.84	0.84	0.59	0.86
ΔTP, /100 msec^2^		0.89^c^	0.90	0.89^c^	0.94	0.90
ΔLF/HF ratio						
AIC	208.8	200.9	202.9	200.9	203.9	202.9
BIC	231.5	230.4	234.6	230.3	233.3	234.6
AUC	0.726^b^	0.804^a^	0.804^a^	0.802^a^	0.801^a^	0.801^a^
P value	0.002	<0.001	<0.001	<0.001	<0.001	<0.001

Abbreviation: DM, diabetes mellitus; CAD, coronary artery disease; CHF, congestive heart failure; UFR, ultrafiltration rate; iPTH, intact parathyroid hormone; ARB, angiotensin II receptor blocker; ACEI, angiotensin-converting-enzyme inhibitor; CCB, calcium channel blocker; HR, heart rate; RRI, R-R interval; SDNN, the standard deviation of N-N interval; RMSSD, the square root of the mean squared differences of successive NN intervals; VLF, very low frequency; LF, low frequency; HF, high frequency; TP, total power; AIC, the Akaike Information Criterion; BIC, the Bayesian Information criterion; AUC, the area-under curve.

Δ represents changes between the early and the middle phase.

Model 1, basic model in which the prior history of DM, CAD, and CHF, age, UFR, iPTH, and the history of antihypertensive agents were incorporated.

^a^Hazard ratio with P < 0.001.

^b^Hazard ratio with P < 0.01.

^c^Hazard ratio with P < 0.05.

## Discussion

IDH is a critical complication associated with the increased mortality in patients with ESRD on hemodialysis^1,2^. These observations have encouraged several studies on preventing IDH^22–25^. Increasing dialysis time and/or frequency could help prevent IDH^26^. However, in the real world, costs and insurance must be considered. Sodium and ultrafiltration profiling, alteration of dialysate composition, and cool dialysate temperature could also help prevent IDH^23^. Several studies were done on early detection of IDH using a photoplethysmograph^27^ and HRV^15,16^. Monitoring in real time during hemodialysis is theoretically a best practice, but it would increase the workload of the nursing staffs and require complex equipment. Given these facts, if intermittent HRV measurement turns out helpful for predicting IDH, it might be able to predict IDH in real-world terms. Our study showed that IDH might be predicted more precisely by measuring HRV just once a month by electrocardiography.

Several characteristic features were noted in Fig. 1. As reported previously, HR is negatively associated with RRI in a nonlinear manner^28^. Interestingly, the change patterns between SDNN and TP (Fig. 1C,H), and between RMSSD and HF (Fig. 1D,G) were similar. These similarities are explained by both mathematical and physiological relationships^13^. As previously reported^29^, the similar pattern between SDNN (Fig. 1C) and TP (Fig. 1H) is also displayed in VLF (Fig. 1E) or LF (Fig. 1F). Our study also identified the changes in HRV during hemodialysis. LF, which represents the sympathetic tone, increased during hemodialysis as known in a previous study^16^ and was maintained until the end of dialysis (Fig. 1F). Although not statistically significant, LF increase in the non-IDH group seemed to be greater than those in the IDH group. Hence, an increasing sympathetic tone during hemodialysis might be associated with the fewer occurrences of IDH. In contrast, RMSSD and HF, the markers of parasympathetic tone, remained unchanged in the IDH group; however, in the non-IDH group, the parasympathetic tone significantly increased and then decreased (Fig. 1D,G). LF/HF ratio is a marker of sympathetic-parasympathetic balance^28^ and significantly increased during hemodialysis in both groups. (Fig. 1I). Although some controversies exist, our results are comparable to those of previous studies^30^, and imply that hemodialysis might improve sympathetic balance by reducing uremic toxins^31^. Our findings also suggest that increases in the LF/HF ratio during hemodialysis may arise from different mechanisms in each group. In the non-IDH group, significant increases in LF increase the LF/HF ratio. However, in the IDH group, this change resulted from a subtle change in HF rather than from a significant increase in LF. Taken together, it appears that autonomic function more easily recovered in the non-IDH group, although there was no significant difference in LF/HF ratio between the groups.

In the univariate model, age was not found to be a significant risk factor, although its *p* value was 0.054. In addition, the prior history of coronary artery disease or congestive heart failure, dialysis vintage, sex, and dialysate temperature were also not found to be significant risk factors. Perhaps this was because ultrafiltration rate is the major contributor to IDH—but the small sample size might also explain that. The use of antihypertensive drug seemed to be protective against IDH in the univariate model. However, undoubtedly, this suggests that the patients in the non-IDH group usually had higher blood pressure compared to the IDH group, rather than a real protective effect against IDH. These results reemphasize the importance of volume status in patients with IDH.

The basic model (Model 1 in Table 4) used only clinical and laboratory information to predict IDH. Although IDH could be predicted using the basic model, when HRV parameters were added, Model 2 showed the best discrimination power to predict IDH (Table 4). Comparison of the model with the highest AUC (Model 2) with the basic model (Model 1) showed that adding HRV parameters to the basic model increased the ability to predict (Fig. 2). The AUC of Model 2 was 0.804 and the degree of increment was statistically significant but not impressive, which suggests that other factors besides autonomic dysfunction affected the development of IDH. The rest portion not explained by our model could probably be explained by the notion of plasma refilling^32^. Unfortunately, to our knowledge, there is no way to assess how much plasma refilling will affect the development of IDH. If the degree to which plasma refilling affects the development of IDH could be quantified or predicted, the model for predicting IDH would become more powerful.

The patients in total received 852 dialysis sessions, and 85 cases (10.0%) of IDH occurred (Table 2). This result is comparable to that from the HEMO Study cohort^8^. Of these, 24 cases recovered when only the Trendelenberg position was used. In seven cases, the hemodialysis had to be terminated early. In this study, K/DOQI criteria was used to define IDH because we hypothesized that autonomic dysfunction is more likely to be closely associated with symptoms with IDH than are those defined by nadir 90, which is defined by systolic blood pressure <90 mm Hg^8^. It was also evaluated whether IDH defined by nadir 90 produced the same results. In fact, it did not.

Our study has several limitations. First, the sample size was small, and the validation was not fully completed in order to make the prediction model. Because it was already recognized that a model based on so few patients and events cannot be used in real clinical practice. We intended this study to show only that using HRV would predict IDH more accurately, even though measuring once in a month. Thus, we focused on demonstrating that using HRV measured monthly could improve the discrimination (AUC) and quality (Akaike Information Criterion and Bayesian Information Criterion), not on trying to build a ‘perfect’ prediction model^33^. Second, the model using HRV showed only whether IDH will occur or not. Thus, future study studies will need to evaluate whether the prevalence of IDH will be reduced when intervention to improve HRV is done.

In conclusion, in the present study, IDH was more precisely predicted by measuring HRV in patients with prevalent hemodialysis. Whether IDH actually declines when HRV is improved and the reproducibility of our methods should be evaluated in further studies. Nevertheless, this study suggests that HRV might be helpful in predicting future IDH.
